# Altered Hippocampal Subfields Functional Connectivity in Benign Paroxysmal Positional Vertigo Patients With Residual Dizziness: A Resting‐State fMRI Study

**DOI:** 10.1111/cns.70175

**Published:** 2024-12-17

**Authors:** Zhengwei Chen, Lijie Xiao, Yueji Liu, Xiue Wei, Zhuo Wang, Xingyi Cao, Haiyan Liu, Yujia Zhai, Liangqun Rong

**Affiliations:** ^1^ Department of Neurology Second Affiliated Hospital of Xuzhou Medical University Xuzhou Jiangsu China; ^2^ Department of Neurology Jinzhou Central Hospital Jinzhou Liaoning China

**Keywords:** benign paroxysmal positional vertigo, fMRI, functional connectivity, hippocampus, residual dizziness

## Abstract

**Objective:**

To explore alterations in functional connectivity (FC) focusing on hippocampal subfields in benign paroxysmal positional vertigo (BPPV) patients with residual dizziness (RD) after successful canalith repositioning procedure (CRP).

**Methods:**

We conducted resting‐state functional magnetic resonance imaging (fMRI) on 95 BPPV patients, comprising 50 patients with RD and 45 without. Seed‐to‐voxel and seed‐to‐seed analyses were employed to examine changes in FC between the two groups. The hippocampal subfields, including the bilateral dentate gyrus (DG), cornu ammonis (CA), entorhinal cortex (EC), subiculum, and hippocampal amygdalar transition area (HATA) were selected as seeds. Additionally, we assessed the relationship between abnormal FC and clinical symptoms.

**Results:**

Seed‐to‐voxel analysis indicated that, compared to non‐RD patients, those with RD exhibited decreased FC between the right DG and right parietal operculum cortex, right HATA and right precuneus, left HATA and left precuneus, left EC and cerebellar vermis 8/−crus 1, and between the left subiculum and left angular gyrus. Conversely, we observed increased FC between the left CA and left lingual gyrus, as well as between the right CA and right fusiform gyrus in RD patients. Furthermore, these variations in FC were significantly correlated with clinical features including the duration of RD and scores on the Hamilton Anxiety Scale and Dizziness Handicap Inventory.

**Conclusion:**

BPPV patients with RD exhibited altered FC between hippocampal subfields and brain regions associated with spatial orientation and navigation, vestibular and visual processing, and emotional regulation. These findings offer novel insights into the pathophysiological mechanisms in BPPV patients with RD following successful CRP.

## Graphical abstract

BPPV patients with RD showed altered FC between hippocampal subfields and brain regions involved in spatial orientation and navigation, vestibular and visual processing, as well as emotional regulation. These findings offer novel insights into the pathophysiological mechanisms in BPPV patients with RD following successful CRP.

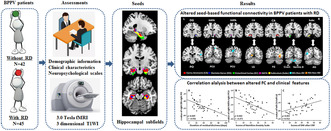

## 1 Introduction

Benign paroxysmal positional vertigo (BPPV), also referred to as otolithiasis, is a peripheral vestibular disorder characterized by recurrent episodes of transient vertigo and distinctive nystagmus triggered by changes in head position relative to gravity [1]. BPPV is recognized as the most prevalent cause of peripheral vertigo, with a lifetime prevalence of 2.4% and an annual prevalence of 1.6% [1, 2]. Canalith repositioning procedure (CRP) is currently regarded as the gold standard treatment for BPPV, as it effectively repositions the displaced otoconial particles from the affected canal back to their original location [3, 4, 5]. However, 29.6%–76.9% of BPPV patients still suffer from a nonspecific sensation of unsteadiness or floating, nonrotating dizziness, disorientation, fogginess, or drowsiness lasting a few days to several months after a successful CRP [6, 7, 8, 9, 10]. These symptoms are called residual dizziness (RD). To date, the pathophysiological mechanisms underlying RD remain unclear.

Recently, resting‐state functional magnetic resonance imaging (rs‐fMRI), a noninvasive neuroimaging technology, has been employed to investigate the pathophysiological mechanisms of RD in patients with BPPV. The rs‐fMRI technique utilizes blood oxygen level dependent (BOLD) imaging to reflect the intrinsic functional activity and connectivity of the brain, proving to be a valuable method for uncovering disease‐induced neural dysfunction associated with neuropathology. This approach has been extensively utilized in scientific research on vestibular disorders, including vestibular migraine (VM) [11], acute unilateral vestibulopathy/vestibular neuritis (AUVP/VN) [12], chronic unilateral vestibulopathy (CUVP) [13], and visually induced dizziness (VID) [14]. Utilizing rs‐fMRI, Fu et al. [15] observed decreased amplitude of low‐frequency fluctuations (ALFF) in the bilateral precuneus in BPPV patients with RD compared to those without RD. Another rs‐fMRI study revealed that BPPV patients exhibited increased fractional ALFF (fALFF) in the bilateral pons and increased regional homogeneity (ReHo) in the left posterior lobe of the cerebellum when compared to healthy controls [16]. The authors indicated that the functional changes in the pons may be closely related to RD following CRP [16]. In our previous work using rs‐fMRI, we confirmed that the occurrence of RD might be associated with decreased functional connectivity (FC) in the parietal operculum cortex 2, increased dynamic network connectivity between the default mode network (DMN) and the visual network (VN), as well as altered internal functional activity in the precuneus and superior temporal gyrus [17, 18, 19].

Similar to persistent postural‐perceptual dizziness (PPPD) and other functional dizziness disorders, BPPV patients with RD often experience impairments in spatial orientation and navigation. The hippocampus is well‐known for its critical roles in these functions [20, 21]. Previous fMRI studies have reported changes in hippocampal functional activity and connectivity in patients with PPPD and chronic subjective dizziness (CSD), indicating impairments in spatial orientation and navigation associated with these conditions [22, 23]. We hypothesized that the pathophysiological mechanism underlying RD involves alterations in FC within the hippocampus. The hippocampus comprises cytoarchitectonically distinct subfields, including the dentate gyrus (DG), cornu ammonis (CA), entorhinal cortex (EC), subiculum, and hippocampal amygdalar transition area (HATA), with each subfield playing a specific role in hippocampal circuitry [24, 25, 26, 27]. However, the FC of these subfields in BPPV patients with RD remains poorly understood. Therefore, utilizing rs‐fMRI, the current study aimed to investigate alterations in FC focused on the hippocampal subfields. Additionally, we sought to determine whether these changes in FC correlate with specific clinical characteristics of BPPV patients with RD following successful CRP.

## 2 Materials and Methods

### 2.1 Participants

Ninety‐five patients diagnosed with BPPV were recruited from the Second Affiliated Hospital of Xuzhou Medical University between May 2020 and June 2024. This cohort included 50 patients with RD and 45 patients without RD. The diagnosis of BPPV was made in accordance with the criteria established by the Committee for Classification of Vestibular Disorders of the Bárány Society in 2015 [28]. The inclusion criteria for BPPV patients were as follows: (1) right‐handed individuals aged 18–75 years; (2) unilateral posterior canal (PC) or horizontal canal (HC) BPPV; (3) patients reporting RD lasting more than 5 days following successful CRP. The exclusion criteria were as follows: (1) patients with recurrent BPPV; (2) patients with BPPV resulting from head trauma; (3) those with anterior semicircular canal or multiple canal BPPV; (4) individuals with concurrent vestibular disorders, such as central vertigo, VM, unilateral peripheral vestibular disorder (UPVD), Meniere's disease (MD), among others; (5) patients with contraindications for MRI examinations; and (6) individuals with end‐stage diseases, severe cardiovascular or cerebrovascular conditions, or severe systemic illnesses. This study received approval from the Ethics Committee of the Second Affiliated Hospital of Xuzhou Medical University ([2020] 021801), and all participants provided informed consent prior to their inclusion in the study.

### 2.2 Procedure

Before enrollment, all patients underwent routine neurological and neuro‐otological examinations. These included neurological and vertigo bedside assessments, vestibular laboratory tests (such as videonystagmography (VNG), video head impulse test (vHIT), vestibular evoked myogenic potentials (VEMP), and rotatory chair tests), audiograms, and otoscopies, as well as conventional MRI examinations (T1WI, T2WI, FLAIR, and DWI). Upon diagnosis, we administered either Epley's maneuver [29] or the Semont maneuver [5] for patients with PC‐BPPV, and the Barbecue rotation maneuver [30] (for geotropic lateral canal) or the Gufoni maneuver [31] (for apogeotropic lateral canal) for patients with HC‐BPPV. Subsequently, all patients underwent a VNG examination—which primarily included the Dix‐Hallpike test [32], a supine‐roll test [33], and a straight head‐hanging test [34]—to assess the success of the CRP. If successful, we collected demographic and clinical data for each patient, including gender, age, education, past medical history (hypertension, hyperlipidemia, diabetes, and coronary heart disease, among others), the involved canal, the affected side, the number of CRP sessions, and the duration of BPPV prior to successful CRP. Furthermore, we evaluated the patients' cognitive levels, anxiety, depression, subjective severity of BPPV, and the impact of BPPV on quality of life using the Montreal Cognitive Assessment Scale (MoCA), Hamilton Anxiety Scale (HAMA), Hamilton Depression Scale (HAMD), vertigo Visual Analog Scale (VAS), and Dizziness Handicap Inventory (DHI), respectively. After successful CRP, all patients underwent a follow‐up assessment on the seventh day, conducted through a face‐to‐face interview. During this assessment, patients were subjected to diagnostic positional tests using VNG. Those exhibiting positional vertigo and nystagmus were excluded from the study. Patients who reported symptoms of RD, including nonrotational dizziness, instability, a sensation of floating, neck tightness, discomfort, and disorientation lasting more than 5 days, were categorized into the RD group. Scores for the DHI and the dizziness VAS were recorded for patients within the RD group after successful CRP. Conversely, patients who did not report any RD symptoms from the time of successful CRP until the follow‐up visit were assigned to the non‐RD group. All patients in both the RD and non‐RD groups underwent rs‐fMRI scanning within the subsequent 2 days. Additionally, the duration of RD symptoms for each patient in the RD group was documented during a later follow‐up, which was conducted weekly via telephone or WeChat over a 3‐month period.

### 2.3 Image Acquisition

Neuroimaging data, including high‐resolution T1‐weighted images (T1WI) and rs‐fMRI, were acquired using a 3.0 T MRI scanner (Discovery MR750; GE Medical Systems, Milwaukee, WI, USA) at the Second Affiliated Hospital of Xuzhou Medical University. During the MRI scanning, all subjects wore noise‐attenuating earplugs and clothing devoid of metal, with their heads stabilized using a sponge cushion. Additionally, all subjects were instructed to close their eyes and maintain stillness of both head and body. They were also asked to remain quiet and relaxed, refrain from engaging in cognitive activities, and avoid falling asleep.

We utilized a three‐dimensional brain volume (3D‐BRAVO) sequence to acquire T1WI data. The scanning parameters were as follows: repetition time (TR) = 2500 ms, echo time (TE) = 3.5 ms, flip angle (FA) = 8°, matrix size = 256 × 256, thickness/gap = 1/0 mm, and number of excitations (NEX) = 1. Additionally, a fast field echo‐planar imaging (EPI) sequence was employed to gather rs‐fMRI data, using the following scanning parameters: TR = 2000 ms, TE = 30 ms, FA = 90°, field of view (FOV) = 200 × 200 mm, matrix size = 64 × 64, thickness/gap = 3.6/0 mm, and a scan duration of 7 min, during which a total of 210 time points were collected for each subject.

### 2.4 Image Preprocessing

The raw data in DICOM format were converted to NIFTI format using the GRETNA software package (https://github.com/sandywang/GRETNA). Subsequently, the first 10 time points were removed by GRETNA to mitigate the impact of initial unstable BOLD signals. The remaining 200 time points underwent preprocessing using the CONN toolbox (Version 18b; http://www.nitrc.org/projects/conn), which is based on the Statistical Parametric Mapping software package (Version 12; http://www.fil.ion.ucl.ac.uk/spm/software/spm12) and operates on the MATLAB platform (Version 2023a; MathWorks Inc., Natick, MA, USA; http://www.mathworks.cn/products/matlab.html). The fMRI data were preprocessed following the default procedures within the CONN toolbox, which included: (1) functional slice‐timing correction to reduce variability in acquisition times across layers during scanning; (2) functional realignment; (3) functional outlier detection, utilizing ART‐based identification of outlier scans for scrubbing, with a head motion threshold set at 3 mm and a global signal threshold at *z* = 9; (4) centering of functional and structural images at coordinates (0, 0, 0); (5) functional segmentation and normalization using DARTEL; and (6) spatial smoothing to enhance image normality, performed with an isotropic Gaussian kernel set at a full‐width at half maximum of 6 mm.

### 2.5 FC Calculation

The preprocessed functional images were subjected to band‐pass filtering (0.01–0.08 Hz) and linear regression to account for confounding effects, including those from white matter, cerebrospinal fluid, and resting state influences. The quality of the denoising procedure was assessed visually using quality assurance report plots generated by the CONN toolbox.

After denoising, a first‐level analysis step was conducted. Seed‐to‐voxel and region of interest (ROI)‐to‐ROI FC were calculated. The 10 subfields of the hippocampus were selected as seeds (ROIs), including the bilateral DG, CA, EC, subiculum, and HATA, which were derived using the template from the Anatomy Toolbox's cytoarchitectonic probabilistic maps [24, 35, 36]. In the seed‐to‐voxel FC analysis, the mean time courses of the 10 seeds were extracted, and Pearson's correlation coefficients (*r*) between these extracted time courses and all other time courses of the whole brain voxels were computed. Subsequently, *r* values were converted into *z*‐scores using Fisher's *r*‐to‐*z* transformation to enhance normality. In the ROI‐to‐ROI FC analysis, a 10 × 10 FC matrix was constructed. A bivariate correlation was employed to calculate the total linear temporal associations among the resulting 45 ROI‐to‐ROI functional connections.

The global signal regression (GSR) is a topic of great debate in rs‐fMRI analyses. Therefore, we also performed an analysis with GSR. The results are displayed in Table S1 and Figure S1.

### 2.6 Statistical Analysis

#### 2.6.1 Analysis of Demographics and Clinical Features

Statistical analyses of demographics and clinical features were conducted using the Statistical Package for the Social Sciences (SPSS, version 24.0; IBM Corp., Armonk, NY, USA). The Skewness and Kurtosis tests for normality were used to assess data distribution. All the quantitative data were subject to tests for normality. Two‐sample *t*‐tests were employed to analyze the differences between the two groups in terms of age, education, duration of BPPV, CRP times, and scores on the HAMA, HAMD, MoCA, as well as the vertigo VAS and DHI scores prior to successful CRP. Additionally, the Chi‐square test was utilized to assess the differences in gender, involved semicircular canal, affected side, hypertension, hyperlipidemia, diabetes, and coronary heart disease between the two groups. A *p*‐value of less than 0.05 was deemed statistically significant.

#### 2.6.2 Analysis of FC

Statistical analyses of rs‐fMRI data were conducted using the CONN toolbox. Two‐sample *t*‐tests were employed to examine the differences in seed‐to‐voxel and ROI‐to‐ROI FC between BPPV patients with and without RD while controlling for gender, age, years of education, and scores on the HAMA, HAMD, and MoCA. The statistical threshold was established at a voxel‐level significance of *p* < 0.001 and a cluster‐level significance of *p* < 0.05, corrected for false discovery rate (FDR), using a two‐tailed approach.

#### 2.6.3 Correlation Analysis

Pearson's partial correlation analysis was performed to investigate the relationships between FC changes and clinical characteristics in BPPV patients with RD. The analysis included variables such as the duration of BPPV, pre‐CRP scores on the VAS for vertigo and the DHI, the duration of RD, and post‐CRP scores on the VAS for dizziness and the DHI, as well as scores on the HAMA and the HAMD. Gender, age, years of education, affected side and semicircular canal, as well as scores on the MoCA were included as covariates. A significance level of *p* < 0.05 was established.

## 3 Results

### 3.1 Demographics and Clinical Features

A total of five patients with RD and three patients without RD were excluded from the study due to significant head motion or inadequate spatial normalization during the fMRI data processing. Consequently, the final statistical analysis included 45 patients with RD and 42 patients without RD. The demographic information and clinical characteristics of the 87 patients are summarized in Table 1. No significant differences were observed between the two groups regarding gender, age, years of education, involved semicircular canal, affected side, number of CRP, hypertension, hyperlipidemia, diabetes, and coronary heart disease, as well as scores on the MoCA, HAMD, and vertigo VAS prior to successful CRP (all *p* > 0.05). However, patients with RD exhibited a longer duration of BPPV (*p* < 0.0001) and higher scores on the HAMA (*p* < 0.0001) and DHI (*p* = 0.004) compared to patients without RD before successful CRP.

**TABLE 1:** Demographic information and clinical features of BPPV patients with and without RD.

	RD (*n* = 45) (Mean ± SD)	Non‐RD (*n* = 42) (Mean ± SD)	*p*
Gender (female/male)	36/9	28/14	0.159
Age (years)	56.51 ± 10.21	52.33 ± 10.48	0.063
Education (years)	9.22 ± 3.20	10.48 ± 4.05	0.112
MoCA	26.69 ± 1.77	27.26 ± 1.48	0.106
HAMA	15.82 ± 4.87	9.83 ± 3.68	< 0.0001
HAMD	8.71 ± 3.71	7.19 ± 4.26	0.101
Involved canal (P/H)	29/16	34/8	0.085
Affected side (L/R)	21/24	15/27	0.300
Hypertension (yes/no)	23/22	14/28	0.094
Hyperlipidemia (yes/no)	22/23	18/24	0.573
Diabetes (yes/no)	12/33	8/34	0.399
Coronary heart disease (yes/no)	9/36	7/35	0.688
Before successful CRP			
Duration of BPPV (days)	12.44 ± 6.40	7.52 ± 3.31	< 0.0001
Number of CRP	6.40 ± 2.76	5.95 ± 2.40	0.423
Vertigo VAS	5.69 ± 1.68	6.26 ± 1.90	0.139
DHI	47.16 ± 13.78	38.00 ± 14.83	0.004
After successful CRP			
Duration of RD (days)	34.36 ± 20.19	NA	NA
Dizziness VAS	3.11 ± 1.28	NA	NA
DHI	27.16 ± 13.78	NA	NA

Abbreviations: BPPV, benign paroxysmal positional vertigo; CRP, canalith repositioning procedure; DHI, dizziness handicap inventory; H, horizontal semicircular canal; HAMA, hamilton anxiety scale; HAMD, hamilton depression scale; L, left; MoCA, montreal cognitive assessment scale; NA, not applicable; P, posterior semicircular canal; R, right; RD, residual dizziness; VAS, visual analog scale.

### 3.2 Results of FC

**FIGURE 1:**
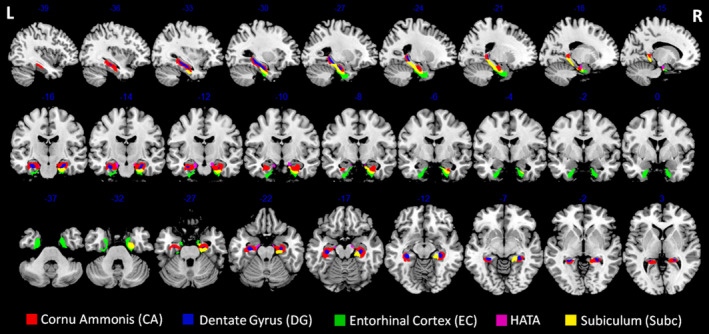
The subfields of the hippocampus. L, left; R, right; HATA, hippocampal amygdalar transition area.

**FIGURE 2:**
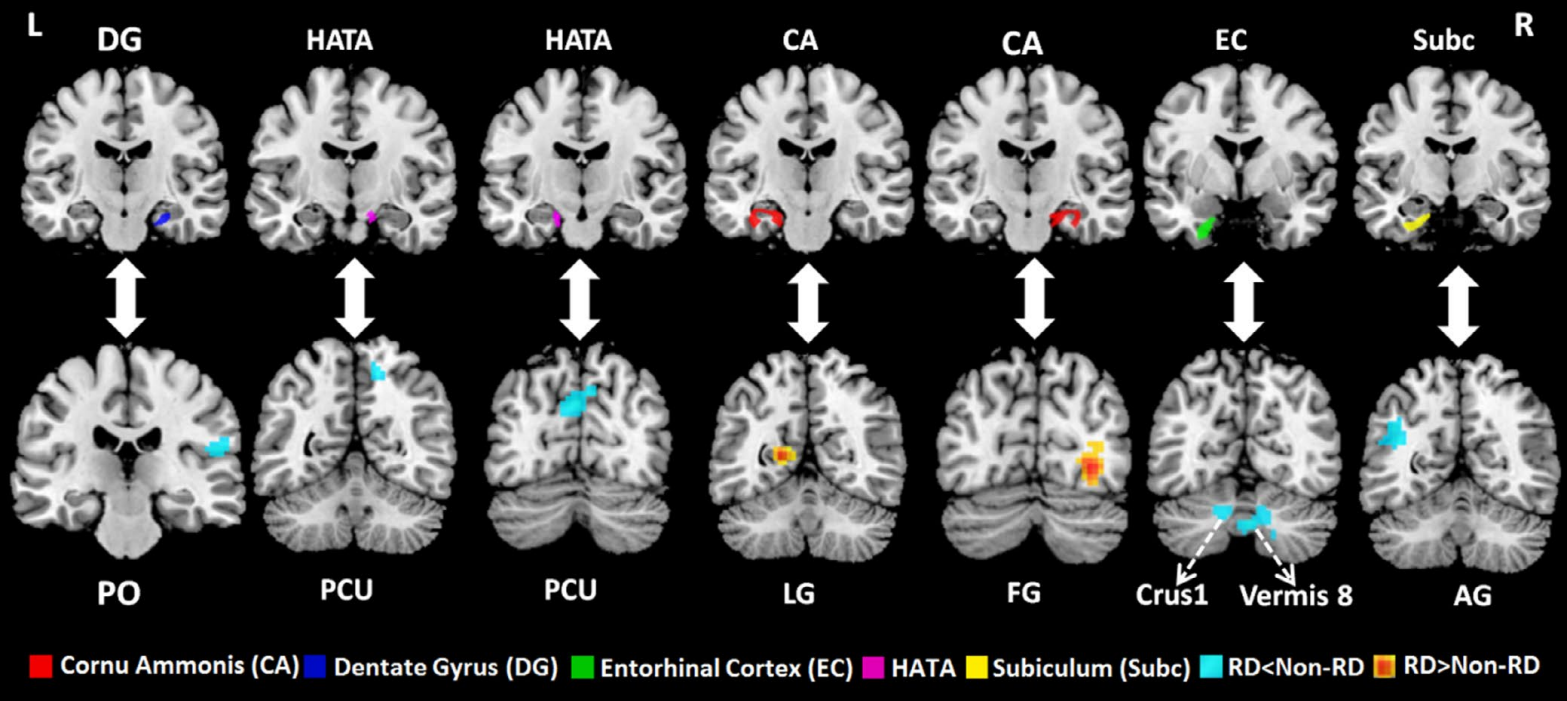
Brain regions with significant differences in seed‐based functional connectivity between BPPV patients with and without RD (voxel‐level *p* < 0.001; cluster‐level *p* < 0.05 [false discovery rate (FDR) correction, two‐tailed]). BPPV, benign paroxysmal positional vertigo; RD, residual dizziness; L, left; R, right; HATA, hippocampal amygdalar transition area; PO, parietal operculum cortex; PCU, precuneus; LG, lingual gyrus; FG, fusiform gyrus; AG, angular gyrus.

**FIGURE 3:**
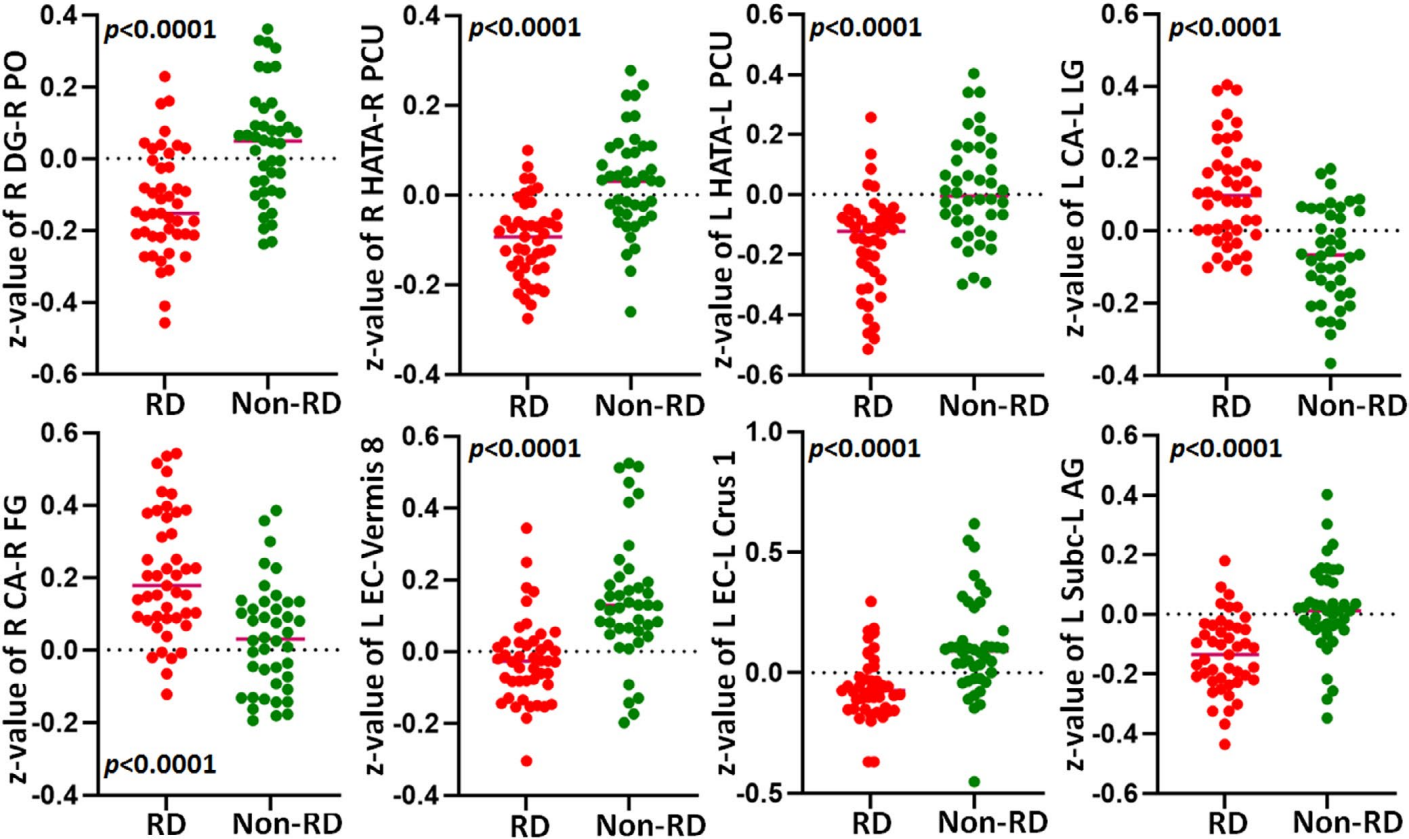
The *z*‐value of BPPV patients with and without RD (all *p* < 0.0001). BPPV, benign paroxysmal positional vertigo; RD, Residual dizziness; L, Left; R, Right; DG, Dentate gyrus; HATA, Hippocampal amygdalar transition area; CA, Cornu ammonis; EC, Entorhinal cortex; Subc, Subiculum; PO, parietal operculum cortex; PCU, Precuneus; LG, Lingual gyrus; FG, Fusiform gyrus; AG, Angular gyrus.

Figure 1 illustrates the subfields of the hippocampus. Using these 10 subfields as seeds, ROI‐to‐ROI analysis revealed no significant differences between BPPV patients with and without RD. In seed‐to‐voxel analysis, patients with RD exhibited decreased FC between the right DG and the right parietal operculum (PO) cortex, the HATA and the right precuneus, the left HATA and the left precuneus, the left EC and the cerebellar vermis 8, the left EC and the left cerebellar crus 1, as well as between the left subiculum and the left angular gyrus (AG) (voxel‐level *p* < 0.001; cluster‐level *p* < 0.05, FDR corrected, two‐tailed), as shown in Table 2, Figures 2 and 3. Additionally, we noted increased FC between the left CA and the left lingual gyrus (LG), as well as increased FC between the right CA and the right fusiform gyrus (FG) in BPPV patients with RD compared to those without RD (voxel‐level *p* < 0.001; cluster‐level *p* < 0.05, FDR corrected, two‐tailed), as depicted in Table 2, Figures 2 and 3.

**TABLE 2:** Brain regions with significant differences in FC between BPPV patients with and without RD.

Seeds	Regions	Peak MNI coordinates *x*, *y*, *z*	Voxel size	Peak *t*	BA
R DG	R PO	57, −24, 18	52	−4.4807	40
R HATA	R PCU	15, −54, 54	40	−4.9092	7
L HATA	L PCU	0, −72, 30	47	−4.3905	18
L CA	L LG	−15, −60, 9	152	5.2395	23
R CA	R FG	30, −75, −3	43	4.6950	19
L EC	Vermis 8	3, −63, −36	57	−4.3488	18
L EC	L Crus1	−15, −69, −6	36	−4.7553	19
L Subc	L AG	−42, −57, 30	40	−4.7523	39

*Note:* Significance was determined at a voxel‐level threshold (*p* < 0.001) and a cluster‐level threshold (*p* < 0.05, two‐tailed) corrected by false discovery rate (FDR).

Abbreviations: AAL, anatomical automatic labeling; AG, angular gyrus; BA, brodmann area; BPPV, benign paroxysmal positional vertigo; CA, cornu ammonis; DG, dentate gyrus; EC, entorhinal cortex; FC, functional connectivity; FG, fusiform gyrus; HATA, hippocampal amygdala transition area; L, left; LG, lingual gyrus; MNI, montreal neurological institute; PCU, precuneus; PO, parietal operculum cortex; R, right; RD, residual dizziness; Subc, subiculum.

In results with GSR, the FC patterns of hippocampal subregions with other brain regions were similar to the above results without GSR. The main difference was that the decreased FC between the HATA and the precuneus, as well as between the left subiculum and the left AG were not observed in results with GSR. Another difference in results with GSR was that the left subiculum showed decreased FC with the left precuneus in BPPV patients with RD compared with patients without RD (voxel‐level *p* < 0.001; cluster‐level *p* < 0.05, FDR corrected, two‐tailed; Table S1 and Figure S1).

### 3.3 The Relationship Between Altered FC and Clinical Features in Patients With RD

**FIGURE 4:**
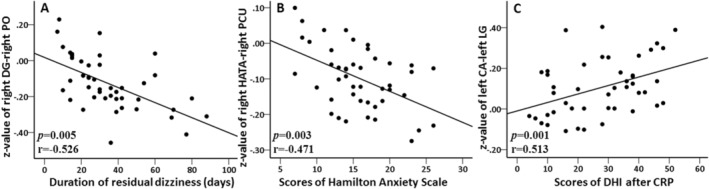
(A) The *z*‐value between right dentate gyrus (DG) and right parietal operculum (PO) cortex was negatively correlated with the duration of residual dizziness (*p* = 0.005, *r* = −0.526); (B) The *z*‐value between right hippocampal amygdalar transition area (HATA) and right precuneus (PCU) was negatively correlated with the scores of Hamilton Anxiety Scale (*p* = 0.003, *r* = −0.471); (C) The *z*‐value between left cornu ammonis (CA) and left lingual gyrus (LG) was positively correlated with the scores of Dizziness Handicap Inventory (DHI) after successful canalith repositioning procedure (CRP) (*p* = 0.001, *r* = 0.513).

Figure 4 illustrates the relationship between altered FC and clinical characteristics in patients with RD. The FC (*z*‐value) between the right DG and right PO cortex showed a negative correlation with the duration of RD (*p* = 0.005, *r* = −0.526). Additionally, the FC (*z*‐value) between the right HATA and right precuneus demonstrated a negative correlation with HAMA scores (*p* = 0.003, *r* = −0.471). Conversely, the FC (*z*‐value) between the left CA and left LG exhibited a positive correlation with DHI scores following successful CRP (*p* = 0.001, *r* = 0.513).

## 4 Discussion

To our knowledge, the present study is the only one that has explored the alterations in resting‐state FC focusing on the subfields of the hippocampus in BPPV patients with RD following successful CRP. The abnormal FC of the hippocampal subfields was primarily observed in the PO cortex, precuneus, LG, FG, cerebellar vermis 8, cerebellar crus 1, and the AG. Furthermore, the altered FC of the hippocampal subfields was found to correlate with specific clinical features of BPPV patients with RD.

In the current study, patients with BPPV who exhibited RD demonstrated a longer duration of BPPV and higher scores on the DHI and HAMA prior to successful CRP, when compared to patients without RD. A recent systematic review and meta‐analysis identified longer duration of BPPV, higher DHI scores prior to treatment, and anxiety as risk factors for RD in BPPV patients following successful CRP [37]. Therefore, our findings align with existing literature, reinforcing the perspective that BPPV patients with prolonged episodes of BPPV, more severe dizziness handicap, and elevated anxiety levels are at an increased risk of developing RD symptoms. Additionally, the duration of RD symptoms among BPPV patients in our study ranged from 7 to 88 days, with a mean of approximately 34 days and a median of 30 days. These results are generally consistent with prior reports in the literature [7, 8, 9].

The human hippocampus is essential for various aspects of memory and plays a crucial role in spatial orientation and navigation [21, 38, 39]. Electrophysiological studies in monkeys have indicated that the hippocampus may be significant for processing spatial information [40]. Previous research involving patients with unilateral hippocampal sclerosis [21], unilateral temporal lobectomy [41], or hippocampal lesions [42] has demonstrated the hippocampus's importance in spatial memory and navigation. Neuroimaging studies utilizing fMRI in healthy subjects have shown hippocampal activation during tasks involving mental imagery of navigation [43], as well as during orientation in virtual interior environments [44]. The hippocampus consists of subfields, including the DG, CA, EC, subiculum, and HATA, with each subfield potentially serving a specific role within the hippocampal circuitry [24, 25, 26, 27].

The DG, a narrow crenated strip of gray matter within the hippocampal circuitry, functions as a “gate” that regulates the flow of information into the hippocampus [45, 46]. It is essential for learning, memory, spatial navigation, and mood regulation [45, 46]. The present study identified decreased FC between the right DG and the right PO cortex in BPPV patients with RD. The PO cortex is recognized as a critical component of the human parieto‐insular vestibular cortex (PIVC) and plays a pivotal role in vestibular processing [47, 48]. Our earlier research has demonstrated changes in FC within the PO cortex in BPPV patients with RD [17]. Therefore, the current study suggests decreased FC between the hippocampus and vestibular cortex, which is involved in spatial navigation and vestibular processing.

The CA can be subdivided into CA1, CA2, CA3, and CA4, which contain place cells that encode location information within the environment and play a critical role in place learning [49, 50]. It is believed that the CA is essential for spatial orientation [49, 50]. The present study identified increased FC between the left CA and left LG, as well as between the right CA and right FG, in BPPV patients with RD. The LG, located in the medial occipital lobe, is necessary for both basic and higher‐level visual processing and is considered a core component of the human visual cortex [51, 52]. The FG is recognized as a key structure involved in processing high‐level visual information, such as face perception and object recognition [52, 53]. Therefore, our results indicate increased FC between brain regions associated with spatial orientation and visual processing in BPPV patients with RD. The findings of the current study further support our previous work, which reported increased FC between the DMN and VN in BPPV patients with RD [18], suggesting that the abnormal FC within the visual cortex network may play an important role in the development of RD.

The vestibular and visual systems are recognized as crucial for precise spatial orientation and navigation [21, 54, 55]. A prior study has indicated the distinct processing of vestibular information in the anterior hippocampus and visual information in the posterior hippocampus [20]. Furthermore, earlier research has proposed a strong connection between the hippocampus and the visual/vestibular cortices [56, 57]. Consequently, we hypothesize that alterations in the FC between the hippocampus and the vestibular/visual cortices may be associated with the spatial orientation and navigation deficits observed in BPPV patients with RD.

The EC is recognized as a critical region for processing and gating spatial information destined for storage in the hippocampus [58]. The cerebellar vermis receives mossy fibers from both vestibular afferents and neurons from the vestibular nuclei and is believed to play a significant role in spatial orientation [44, 59]. The cerebellar crus 1 interacts with the hippocampus and is suggested to be involved in spatial orientation [60, 61]. The subiculum, which serves as the primary output structure of the hippocampus, plays a crucial role in spatial orientation and navigation due to the presence of head direction cells and grid cells [62, 63, 64]. The AG, situated in the posterior part of the inferior parietal lobule, has been reported to be involved in spatial navigation and the processing of spatial information [65, 66]. Therefore, the decreased FC between the EC and the cerebellar regions, as well as between the subiculum and the AG in RD patients potentially indicate a reduction in FC in brain regions that are critical for spatial orientation and navigation.

The HATA serves as a transition zone between the head of the hippocampus and the tail of the amygdala. The present study identified a decrease in FC between the HATA and the precuneus. Previous research has indicated that the volume of the HATA is associated with perinatal stress, posttraumatic stress disorder, and depression [67, 68, 69]. Altered hippocampal dynamic FC was reported to be related to bipolar disorder with suicidal attempt [70]. Additionally, the functional activity of the HATA may correlate with the severity of depression in major depressive disorder [71]. Collectively, these studies suggest that the HATA plays a significant role in emotional regulation. Prior investigations have established a robust connection between the precuneus and emotional disorders such as anxiety and depression, indicating that the precuneus is involved in emotional regulation [72, 73, 74]. Consistent with the findings of the current study, ab normal functional activity of the precuneus has been previously reported in patients with RD [15, 19]. Anxiety is closely associated with RD and is recognized as a primary risk factor for its occurrence [37, 75]. The RD patients included in this study exhibited significant anxiety. We propose that the decreased FC between the HATA and the precuneus is linked to impairments in emotional regulation among BPPV patients with RD.

Insights emerging from mapping intrinsic brain connectivity provide a potentially mechanistic framework for an understanding of aspects of human behavior and the pathophysiology of neurological and psychotic disorders [76, 77, 78, 79]. The current study explored the possible pathophysiological mechanism of RD by mapping the intrinsic connectivity of hippocampal subregions in patients with RD. The identified hippocampal FC markers of RD potentially have an important enlightening effect on the prevention and treatment of BPPV patients with RD.

## 5 Limitations

However, the present study has several potential limitations. First, the sample size is relatively small, necessitating future studies with a larger number of BPPV patients, both with and without RD, to enhance the generalizability of our findings. Second, despite the complex functional and structural connections among hippocampal subfields [80, 81], the ROI‐to‐ROI FC analysis in this study did not identify abnormal FC among these subfields in patients with RD. Third, certain subregions of the hippocampus, such as the hippocampal fimbria and the hippocampal fissure, were excluded from the FC analysis. Fourth, Pearson's partial correlation analysis in the current study was not corrected by multiple comparison correction. Fifth, we did not calculate the frame‐wise displacement (FD) during fMRI data processing. Another potential limitation is that BPPV patients with anxiety or depression were not excluded from the current study. Lastly, BPPV patients with RD were not assessed using tasks related to spatial orientation and navigation, which could further validate the results of the current study.

## 6 Conclusion

In summary, patients with BPPV who exhibited RD demonstrated altered FC between hippocampal subfields and brain regions associated with spatial orientation and navigation, vestibular and visual processing, as well as emotional regulation. These findings offer novel insights into the pathophysiological mechanisms underlying RD in patients with BPPV following successful CRP.

## Author Contributions

Zhengwei Chen designed the study, analyzed the data, and wrote the main manuscript. Lijie Xiao and Yueji Liu collected the data. Xiue Wei and Xingyi Cao organized the data. Zhuo Wang searched the literature. Haiyan Liu, Yujia Zhai, and Liangqun Rong revised the manuscript. All authors contributed to the article and approved the submitted version.

## Ethics Statement

This study received approval from the Ethics Committee of the Second Affiliated Hospital of Xuzhou Medical University, and all participants provided informed consent prior to their inclusion in the study.

## Conflicts of Interest

The authors declare no conflicts of interest.

## Supporting information


Data S1.

## Data Availability

The data that support the findings of this study are available on request from the corresponding author. The data are not publicly available due to privacy or ethical restrictions.
