# Supplementary material for: Altered Hippocampal Subfields Functional Connectivity in Benign Paroxysmal Positional Vertigo Patients With Residual Dizziness: A Resting‐State fMRI Study
Source: CNS Neurosci Ther. 2024 Dec 17;30(12):e70175. doi: 10.1111/cns.70175 (PMC11652785; doi:10.1111/cns.70175)
Supplement: Supplementary file 1 — Data S1. [file CNS-30-e70175-s001.docx]

Supplementary Material

# Supplementary Table

**Table S1** Brain regions with significant differences in FC between BPPV patients with and without RD (with global signal regression)

| Seeds | Regions | Peak MNI coordinates  x, y, z | Voxel size | Peak t-value | BA |
| --- | --- | --- | --- | --- | --- |
| R DG | R PO | 56 -21 19 | 39 | -4.4793 | 40 |
| L CA | L LG | -11 -58 3 | 110 | 5.2007 | 18 |
| R CA | R FG | 30 -76 -5 | 65 | 4.5877 | 19 |
| R CA | R FG | 42 -39 -15 | 31 | 4.3102 | 37 |
| L EC | L Crus1 | -30 -72 -30 | 36 | -4.2290 | 19 |
| Subc | L Precuneus | -6 -51 15 | 40 | -4.1657 | 23 |

**Abbreviations:** Significance was determined at a voxel-level threshold (*p*<0.001) and a cluster-level threshold (*p*<0.05, two-tailed) corrected by false discovery rate (FDR)；FC, Functional connectivity; BPPV, Benign paroxysmal positional vertigo; RD, Residual dizziness; MNI, Montreal neurological institute; BA, Brodmann area; L, Left; R, Right; DG, Dentate gyrus; CA, Cornu ammonis; EC, Entorhinal cortex; Subc, Subiculum; PO, parietal operculum cortex; LG, Lingual gyrus; FG, Fusiform gyrus.

# Supplementary Figure


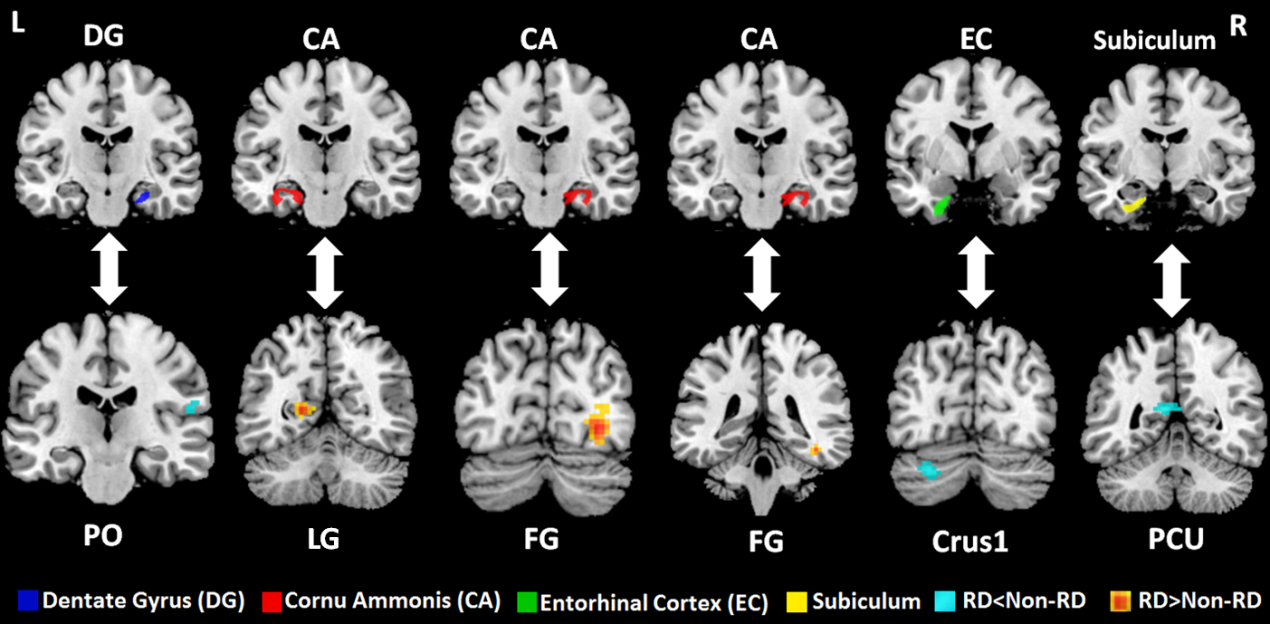


**Fig. S1** Brain regions with significant differences in seed-based functional connectivity between BPPV patients with and without RD (with global signal regression; voxel-level *p*<0.001; cluster-level *p*<0.05 (false discovery rate (FDR) correction, two-tailed)). BPPV, Benign paroxysmal positional vertigo; RD, Residual dizziness; L, Left; R, Right; PO, parietal operculum cortex; LG, Lingual gyrus; FG, Fusiform gyrus; PCU, Precuneus.
